# Supplementary material for: Robust model-based analysis of single-particle tracking experiments with Spot-On
Source: eLife. 2018 Jan 4;7:e33125. doi: 10.7554/eLife.33125 (PMC5809147; doi:10.7554/eLife.33125)
Supplement: Supplementary file 1. [file elife-33125-supp1.pdf]

Spot-On Manual (v0.10)

An updated version of this manual is online on
`https://spoton.berkeley.edu/SPTGUI/docs/latest`

Authors:
Anders S. Hansen*
Maxime Woringer*
Jonathan B. Grimm
Luke D. Lavis
Robert Tjian
Xavier Darzacq

December 20, 2017

*these authors contributed equally and are alphabetically listed

Abstract

Spot-On allows you to analyze single particle tracking datasets. Spot-On fits a realistic kinetic model to the jump length distribution of the observed trajectories and provides estimates of the fraction bound (F) and diffusion coefficients (D) for either a two state (*bound-free*) or a three state (*bound-free1-free2*) model.

Spot-On is a libre/open-source software and exists both as a web-application and a command-line version.

This project owes a lot to Davide Mazza, who initially developed the conceptual framework implemented in Spot-On (see [Mazza et al., 2012]).

Contents

1 The problem **3**
- 1.1 Motion blur . . . 4
- 1.2 Ambiguous tracking . . . 4
- 1.3 Particles move out of focus . . . 5

2 Quickstart/tutorial **8**
- 2.0.1 Step 1: start an analysis with demonstration files . . . 8
- 2.1 About the demonstration files . . . 9
- 2.2 Overview of the application . . . 10
- 2.3 Import . . . 11
  - 2.3.1 Step 2: rename and tag the uploaded files . . . 12
- 2.4 Quality check . . . 13
  - 2.4.1 Step 3: Inspect a few quality metrics . . . 13
- 2.5 Kinetic modeling . . . 14
  - 2.5.1 Overview of the kinetic modeling tab . . . 14
- 2.6 Computation of the jump length distribution . . . 16
  - 2.6.1 Step 4: compute the empirical jump length distribution for the Sox2 datasets . . . 16
  - 2.6.2 Step 5: play with the display options . . . 20
  - 2.6.3 Step 6: compare the H2B and Sox2 jump length distributions . . . 21
  - 2.6.4 Step 7: mark one jump length distribution for download 22
- 2.7 Model fitting . . . 23
  - 2.7.1 Step 8: fit a two-state model to the H2B data . . . 23
  - 2.7.2 Step 9: mark the plot for download . . . 28
  - 2.7.3 Step 10: fit the Sox2 dataset with a two-state model . 28
  - 2.7.4 Step 11: fit a three-state model . . . 29
  - 2.7.5 Step 12: compare the two-state fits of H2B and Sox2 datasets . . . 30
- 2.8 Download . . . 31
  - 2.8.1 Step 13: download the marked analyses . . . 31

3 Online sections **33**

1 The problem

Within a cell, a DNA-binding factor diffuses and occasionally binds to DNA or forms complexes. Each of these states can be macroscopically characterized by an apparent diffusion coefficient and a fraction of the total population residing in this state. Thus, we are interested in extracting those parameters for each state (Figure 1). Note that even when the observed molecules are stably bound to DNA, they will still exhibit a nonzero diffusion coefficient (reflecting a mixture of the slow motion of chromatin (estimated to be around 0.01-0.02 $\mu m^2/s$, [Shinkai et al., 2016] –, the motion of the cell itself, microscope drift and possibly other factors).

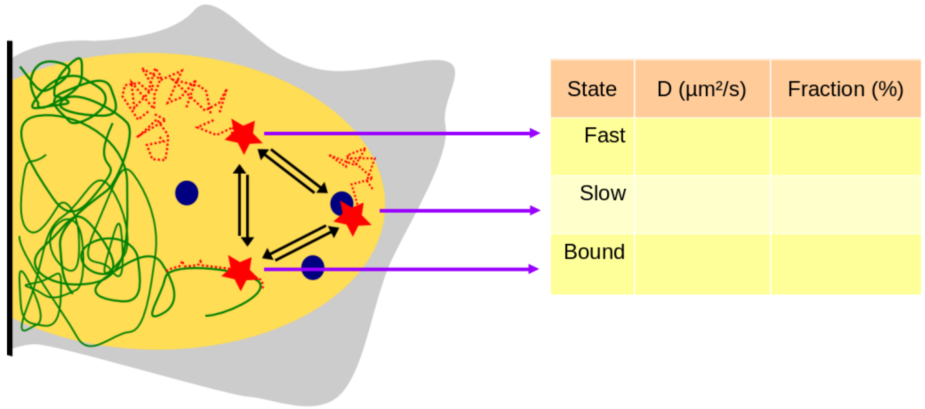

Figure 1: Schematic of a cell with diffusing particles.

To infer those parameters, *single particle tracking* (SPT) approaches can be implemented. In single particle tracking of nuclear proteins, cells are typically engineered to express a protein of interest either fused to a fluorescent protein or to a tag that can be conjugated to a synthetic dye (e.g. HaloTag). When the density of dyes in the focal plane is sufficiently low (because the number of expressed proteins is low, because the depth of field is extremely small or because only a fraction of the molecules are visible at a time), individual molecules appear as isolated spots that can be localized with a subpixel accuracy by fitting a 2D (usually Gaussian) function and performing tracking between successive frames. This yields a series of trajectories, each corresponding to the motion of a single protein-conjugated fluorophore.

Although extremely powerful, single particle tracking of nuclear factors is subject to several methodological difficulties detailed below:

1.1 Motion blur

When a diffusing particle is observed, it will keep diffusing while one frame is acquired. In this case, particles exhibit "motion blur", that is that the photons emitted by a fast-diffusing molecule appear spread across a higher surface than bound molecules. This has several consequences:

- First, fast-diffusing molecules show a reduced signal-to-noise ratio
- Second, these detections significantly deviate from the theoretical PSF (point-spread function) of bound molecules.

Because of these two effects, fast-diffusing particles are harder to detect, especially if PSF-fitting localization algorithms are used. Furthermore, because bound molecules are not affected by motion blur, molecules in the bound state tend to be overestimated because the fast-diffusing molecules are undercounted.

The picture below (Figure 2) shows one frame containing two particles, one immobile particles appear as a very identifiable, Gaussian and symmetric spot (right red spot) whereas the fast-diffusing particle on the left is much harder to detect and very poorly resembles a point-emitter (spread out, left red spot).

Because motion blur results in under-detection of fast-diffusing particles, the amount of missed particles strongly depends on internal settings of the detection algorithm, and cannot readily be corrected after the acquisition. Section "How to acquire a dataset" details a few ways to circumvent these biases at the acquisition step.

In brief, the effect of motion blur can be mitigated by reducing the excitation pulse duration (to minimize the motion of the fast-diffusing population during one exposure) and the laser intensity (to keep the signal-to-noise sufficient).

1.2 Ambiguous tracking

As single particle tracking is intrinsically a low-throughput method, one may want to increase the density of tracked particles per frame in order to accelerate the data collection rate. However, as the density of particles increases,

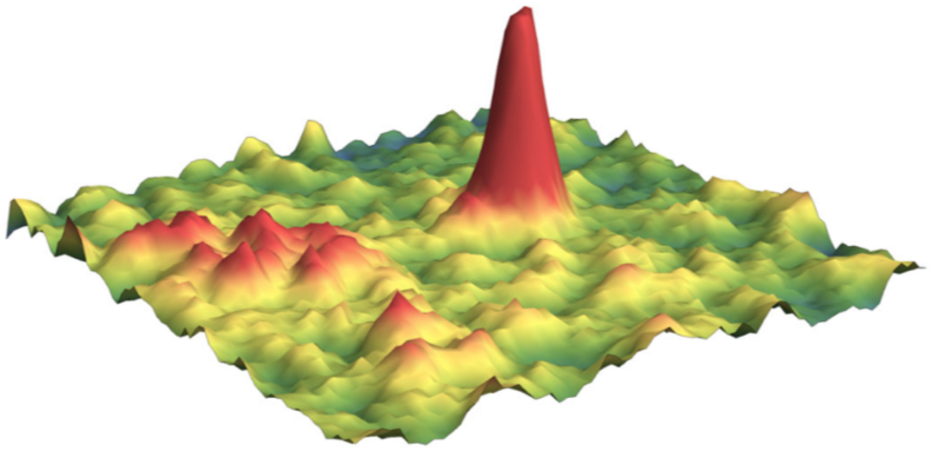

Figure 2: Particles observed with motion blur.

the tracking can become ambiguous. Furthermore, fast-moving particles are again more likely to be misconnected with other unrelated detections. This might result in a truncated jump length distribution, and thus a wrong estimation of the diffusion coefficient.

When imaging with a high density of particles, the nearest detection in the next frame might not be the same particle. In the limit of particles with high diffusion coefficients, it is likely that particles will "cross" each other and that one particle with be connected with another particle.

In practice, this leads to an under-detection of long jumps, because when a particle exhibits a long jump, the tracking algorithm is likely to pick another particle closer in space. This effect results in an underestimation of the fast-diffusing fraction and can be reduced by imaging at a low number of particles per frame. Section "How to acquire a dataset" details a few ways to circumvent those biases at the acquisition step.

1.3 Particles move out of focus

In addition to motion blur biases, that leads to fast-moving particle to be missed by the detection algorithm, particles diffuse out of the detection volume (usually a slice of ~ 1 μm thickness). This effect is virtually zero for bound molecule, but becomes significant for fast-moving particles, leading to an undercounting of this population. The graph below (Figure 3) shows the

jump length distribution of a molecule appearing in two states with respective diffusion coefficients D_1 and D_2 (expressed in μm^2/s).

More precisely, the graph below displays the theoretical jump length distribution in case of an unlimited depth of field (solid line) and the simulation of the observed jump length distribution (dotted line) when particles are only observed within the depth of field of the objective (here set to 0.75 μm, see the FAQ for a method to measure it).

An interactive figure is available on `https://spoton.berkeley.edu/SPTGUI/docs/latest`. This figure features cursors under the simulation to tune the diffusion coefficients of the two populations (D_1 and D_2) and the proportion of the first population (p). From this graph, it appears (1) that as one increases the second diffusion coefficient (D_2), the discrepancy between the solid line and the dotted line increases, reflecting the fact that fast-diffusing particles tend to be under-counted in the observation through a setup with a finite depth of field.

In addition to D_1, D_2 and p, this interactive graph allows you to play with the effect of the localization error σ and the exposure time Δt. Note that this simulation does not take into account motion blur, so the undercounting of fast-diffusing particles is likely to be an underestimate.

Briefly, a reduced exposure time leads tends to limit the fraction of fast-diffusing particles moving out-of-focus from one frame to another. On the other hand, when the frame rate becomes too high, the detections are dominated by the localization error and inference become less and less accurate. Thus, a trade-off between the exposure time and the fast-diffusion coefficient has to be found.

From this representation, one can derive the fraction of particles that will move out of focus in the next frame as a function of the fast-diffusion coefficient and the exposure time (Figure 4; in this case, allowing one gap so that a particle out of focus for one frame can still be reconnected in the following frame).

This graph shows that fast-diffusing molecules ($D > 5\mu m/s$) are extremely hard to track, even at a relatively high frame rate. For instance, when imaging at 100 Hz (10 ms per frame) a factor moving at 10 μm^2/s (such as Halo-3xNLS), 40% of the particles move out of focus at each frame. This drastically limit the number of trajectories coming from the free population.

Furthermore, this graph only represents the fraction of particles remaining in focus after one frame. To get longer trajectories (more than two

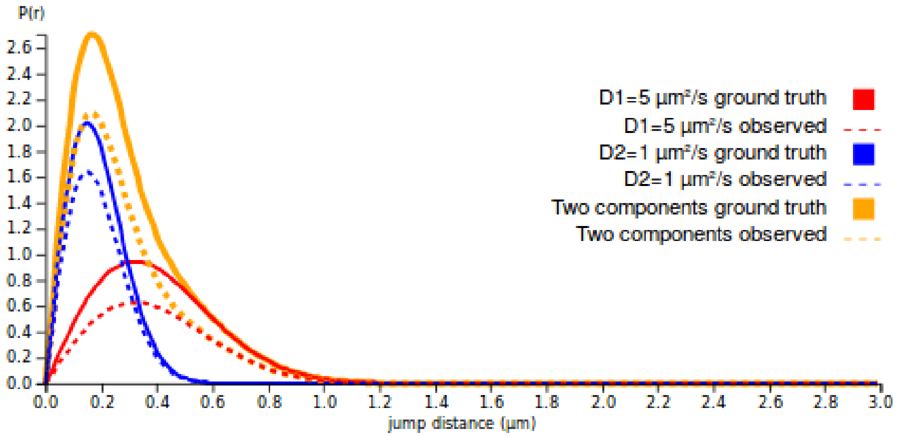

Figure 3: Theoretical jump length distribution.

timepoints) is much harder, and is both limited by photobleaching and particles moving out of focus (detailed in section "What limits the length of trajectories?".

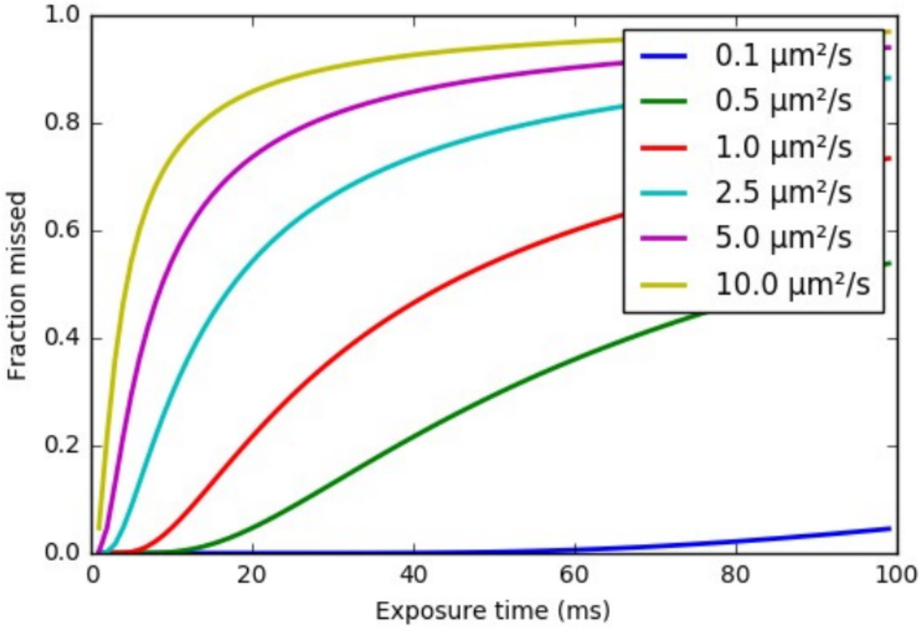

Figure 4: Fraction of particles missed as a function of Δt.

2 Quickstart/tutorial

This section of the Spot-On documentation will guide you through a sample analysis with a couple of demonstration files and will provide you with an overview of Spot-On features and options.

2.0.1 Step 1: start an analysis with demonstration files

To access the demonstration files, go to the Spot-On homepage (`https://spoton.berkeley.edu/`) and scroll to the "Get Started section" (or alternatively click the "Start spotting!" button on the top menu. First fill in the "I'm not a robot" CAPTCHA. Then you have the option to either upload your own tracking files and start your analysis or start with demo files. We will use the demo files for the purpose of this tutorial.

This option will load the analysis page and will automatically import some demonstration file. Also, a custom and permanent URL is created. Your analyses will accessible from this URL until you choose to delete them. Do not share this URL if you want your datasets and analyses to remain

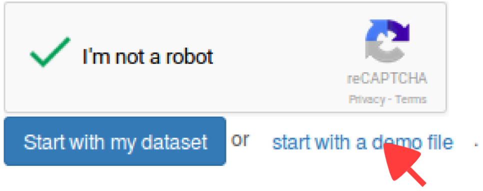

Figure 5: Screenshot of the CAPTCHA region.

private. You might want to bookmark it in order to reaccess the data later. Note that if you lose this address, there is no way for you to recover your files, since your upload is totally anonymous (we do not collect your identity or email address).

2.1 About the demonstration files

When you click on the "Start with a demo file" button, ten sample datasets are loaded. They are part of a bigger dataset described in details in the Datasets section that include single particle tracking of four nuclear proteins: histone H2B (H2B), Sox2, HaloTag-3xNLS and CTCF. These four proteins were imaged through a range of conditions, leading to 1064 cells imaged in total.

By default, the ten imported files are five replicates of histone H2B (fused to both a HaloTag and a SNAP-tag in U2OS cells, labeled with the Halo-PA-JF646 dye and imaged at 74 Hz (that is 1000/74 = 13.5 ms per frame).

The five other files are five replicates of the transcription factor Sox2 (fused to a HaloTag in mouse embryonic stem cells and imaged in comparable conditions: labeled with PA-JF646 and imagied at 74Hz.

In this demo dataset, one of the goal is to get an idea of the dynamics of the Sox2 transcription factor. Indeed, an estimate of the fraction bound and diffusion coefficient of Sox2 provides a valuable insight into how this transcription factor regulates transcription. For instance, a low fraction bound and a high diffusion coefficient could suggest a highly dynamic regulation, but also a target search mechanism dominated by free diffusion. The H2B samples are provided as a reference for a protein that is known to be mostly bound to chromatin, in order to facilitate comparisons with more characterized systems.

2.2 Overview of the application

First of all, Spot-On is organized into *four successive tabs*. These tabs are populated one after the other (that is, for instance, the “Kinetic modeling” tab remains blank as long as no dataset has been uploaded in the “Data” tab, etc). The four tabs are detailed in Table 2.2 ((1) in Figure 6).

Tab	Description
Data	This tab allows you to upload your datasets in various formats in a batch mode, to annotate them, and to see statistics both for individual datasets and for the ensemble of uploaded files.
Kinetic modeling	Performs the fit of the kinetic model according to specified parameters, display the jump length distribution and the corresponding fit. Allows to include or exclude files for analysis. Display and fits can be marked for download.
Download	This tab allows you to download the files marked for download in various formats (PDF, SVG, EPS, PNG, and ZIP archive). The ZIP archive contains the raw data, the fitting parameters and the fitted coefficients.
Settings	Allows you to erase the analysis (together with all the uploaded datasets).

The “Upload dataset” region ((2) in Figure 6), where you can upload from various file formats. Clicking on any of the format will display a box where you can enter additional upload parameters, and will ultimately display a drag-and-drop upload box. Accepted formats are described in more details in the “Input formats” section online.

For the purpose of this tutorial, the data has already been loaded, so we won’t play with this part of the page.

The “Uploaded datasets” region ((3) in Figure 6), that displays the uploaded datasets, together with their status (`uploading`, `queued`, `error`). The meaning of the descriptors in the “status” column in the upload box is detailed in the “Descriptors of imported datasets” section online. When clicking on the “eye” symbol next to an uploaded dataset will display some statistics in the area (4). The meaning and details of the computation of each statistic is detailed in section “Dataset statistics” below. Finally, area (5) displays similar statistics as area (4), but for all the datasets pooled together.

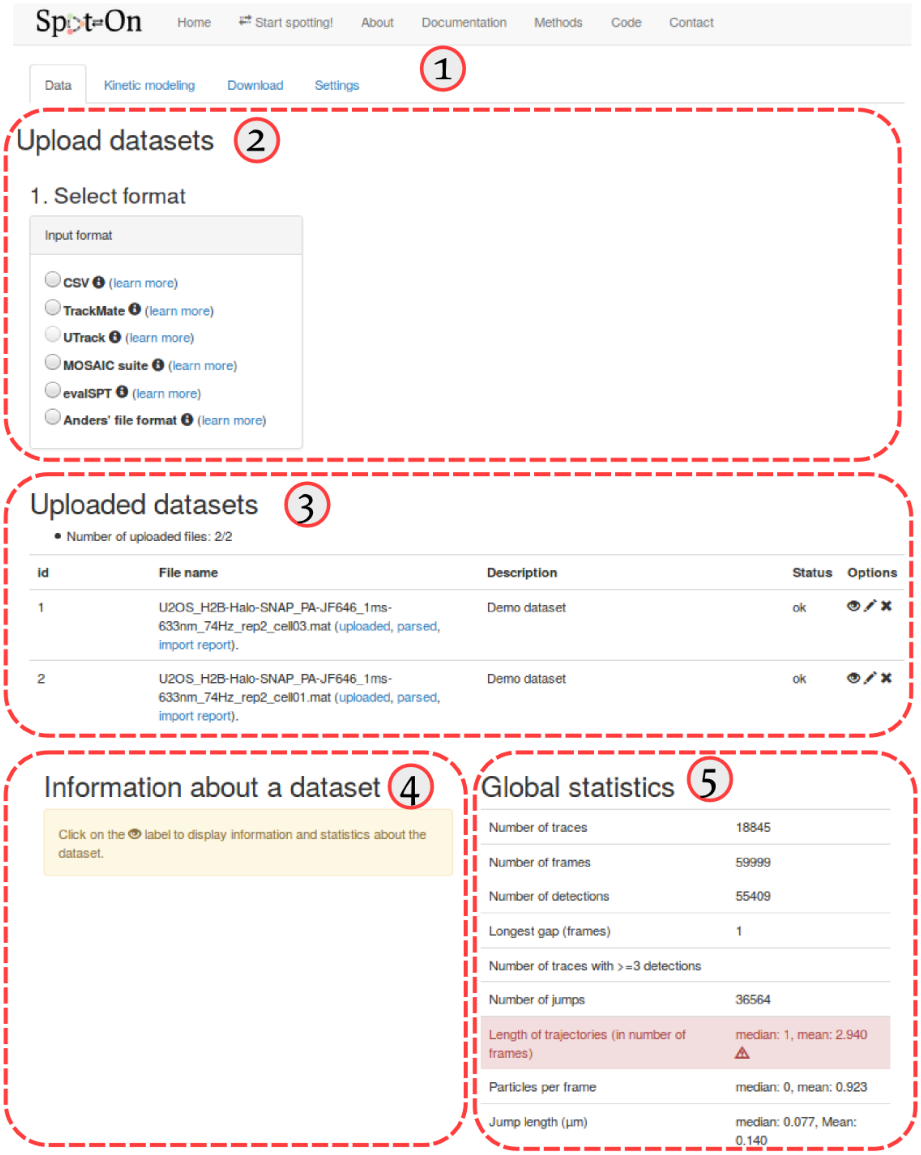

Figure 6: Screenshot of 'Data' tab.

2.3 Import

To proceed with the tutorial, several files have been loaded, they are named. They might get imported in a different order:

1. mESC_C3_Halo-Sox2_PA-JF646_1ms-633nm_74Hz_rep2_cell01.mat
2. mESC_C3_Halo-Sox2_PA-JF646_1ms-633nm_74Hz_rep2_cell02.mat
3. mESC_C3_Halo-Sox2_PA-JF646_1ms-633nm_74Hz_rep2_cell03.mat
4. mESC_C3_Halo-Sox2_PA-JF646_1ms-633nm_74Hz_rep2_cell04.mat
5. mESC_C3_Halo-Sox2_PA-JF646_1ms-633nm_74Hz_rep2_cell05.mat
6. U2OS_H2B-Halo-SNAP_PA-JF646_1ms-633nm_74Hz_rep2_cell01.mat
7. U2OS_H2B-Halo-SNAP_PA-JF646_1ms-633nm_74Hz_rep2_cell02.mat
8. U2OS_H2B-Halo-SNAP_PA-JF646_1ms-633nm_74Hz_rep2_cell03.mat
9. U2OS_H2B-Halo-SNAP_PA-JF646_1ms-633nm_74Hz_rep2_cell04.mat
10. U2OS_H2B-Halo-SNAP_PA-JF646_1ms-633nm_74Hz_rep2_cell05.mat

These files correspond to a subset of an experimental series spanning 1500 cells in several conditions for various transcription factors and DNA-binding proteins, acquired at various framerates and durations of stroboscopic illumination. This dataset is described in more details in the "Datasets" section.

Five of these correspond to the transcription factor Sox2, which has been endogeneously tagged with a HaloTag and observed with the PA-JF646 organic dye [Grimm et al., 2015]. The five other correspond to the Halo-tagged histone H2B imged under the same conditions.

2.3.1 Step 2: rename and tag the uploaded files

Since the naming convention of these files is a little bit cumbersome, let's first edit the description of each file to make it clearer. To do so, click on the "pencil" icon (see (6)) next to each uploaded dataset. An "edit" box will appear at the bottom of the "Uploaded datasets" area, and we can now either rename or add a more explicit description of the datasets. We choose to leave the name as is, but add a short description for each dataset, such as "H2B cell1", "H2B cell2", etc. (7).

> The uploaded dataset comprises two distinct proteins, and five replicates for each protein. In the next steps, we want to make sure that we pool the replicates of each protein together, but do not mix up the two proteins.

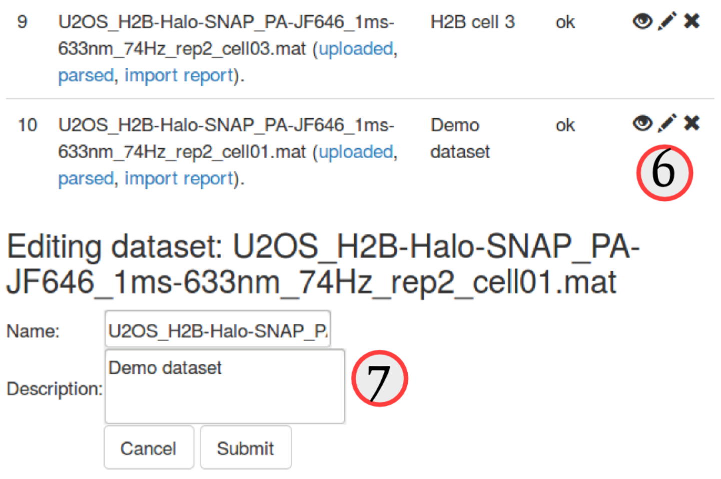

Figure 7: Screenshot: Editing the description of datasets.

2.4 Quality check

Now that we see a little bit clearer through the datasets, let's inspect a little bit the datasets, and try to assess the quality of the dataset. Spot-On provides a few quality metrics (statistics), accessible for each dataset by clicking the "eye" button.

2.4.1 Step 3: Inspect a few quality metrics

Click on the "eye" button next to the datasets and have a look at the metrics displayed. Make sure you familiarize yourself with those.

The table below (Table 2.4.1) summarizes the statistics computed for the first dataset (named `mESC_C3_Halo-Sox2_PA-JF646_1ms-633nm_74Hz_rep2_cell01.mat`).

Statistic	Value
Number of traces	6103
Number of frames	29997
Number of detections	15692
Longest gap (frames)	1
Number of traces with > 3 detections	1813
Number of jumps	9589
Length of trajectories (in number of frames)	median: 1, mean: 2.571
Particles per frame	median: 0, mean: 0.523
Jump length (μm)	median: 0.126, Mean: 0.236

Although the number of jumps is not extremely high, we need to keep in mind that we plan to pool this dataset with four other datasets, which should overcome the limited size of this dataset. In case we encounter a dataset of unsuitable quality, we can exclude it by clicking the "cross" button next to the dataset.

Once that we are confident about the quality of the uploaded data, we can proceed to the second tab, the "Kinetic modeling".

2.5 Kinetic modeling

2.5.1 Overview of the kinetic modeling tab

The "kinetic modeling" tab is divided in several sections (Table 2.5.1 and Figure 8).

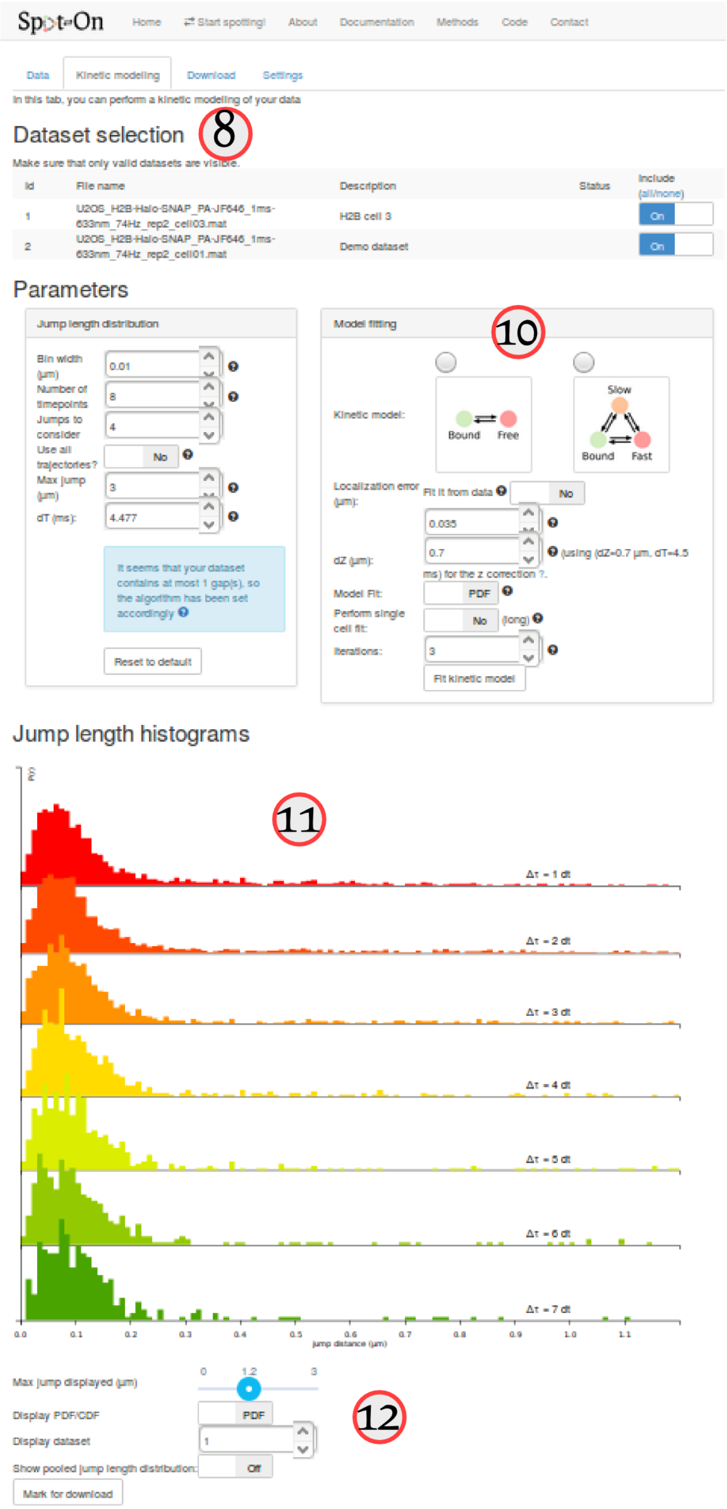

Figure 8: Overview of the kinetic modeling tab.

#	Section	Description
(8)	Dataset selection	This section lists all the uploaded datasets. For each fit of the model, you can choose whether to include one specific dataset for fitting or not.
(9) and (10)	Parameters	Parameters used to compute the empirical jump length distribution (9) and to fit it (9). This includes the choice of a 2-state vs. a 3-state model, the range of the tested parameters, etc.
(11) and (12)	Jump length histogram	This area contains the plot of the jump length distribution, overlaid with the fitted model (if evaluated). It also contains the option to either visualize single datasets or the pool of the selected datasets. Finally, it contains an option to save an analysis for download.

2.6 Computation of the jump length distribution

For the purpose of this tutorial, we'll simply fit the H2B and Sox2 datasets separately, and compare the two-state and three-state models based on their goodness of fit (assessed by the Bayesian Information Criterion, BIC).

2.6.1 Step 4: compute the empirical jump length distribution for the Sox2 datasets

First, in the "Dataset selection" select the five Sox2 replicates. This is done by switching the "Include" toggle button to "On" next to the Sox2 datasets. Make sure that none of the H2B datasets are included (Figure 9).

We can then set the parameters to compute the jump length distribution (Figure 10). We will mostly leave the parameters as default. Section "Jump length distribution computation parameters" describe the role of each parameter in more details.

Then click the '*Compute!* button. After a few seconds, the jump length distribution is computed for all the datasets and appears under the "Jump length distribution" section.

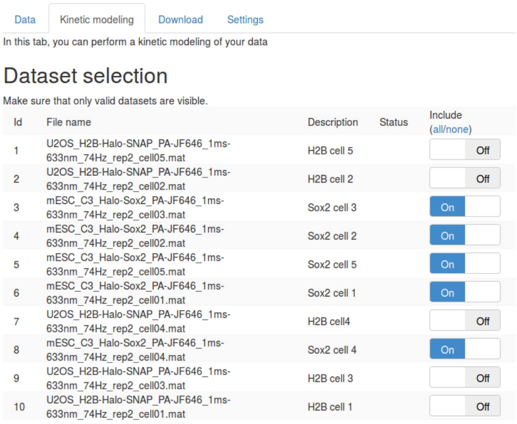

Figure 9: Selection of Sox2 datasets.

The table below (Table 2.6.1) summarizes some key principles to properly set those parameters.

Parameter	Value	Default?	Comment
Bin Width (μm)	0.01	Y	The size of the bin used to build the empirical histogram of jump lengths.
Number of gaps allowed	1	Y	The number of gaps allowed by the tracking algorithm. This has to match the maximum number of gaps allowed by the tracking algorithm.
Number of timepoints	8	Y	The number of Δt to consider when fitting the model. Usually, higher values provide better results, provided that the histogram are sufficiently populated.
Jumps to consider	4	Y	The number of jumps per trajectory actually used to build the histogram. This is empirically useful to correct for overcounting of slow-molecules not accounted for by the corrections implemented in the algorithm (for instance for undercounting due to motion-blur). Here, for each trajectory, the first 4 jumps for each Δt (if possible) will be used to build the jump length histogram. For example, if Number of timepoints=8 and JumpsToConsider=4, a trajectory of 9 frames will contribute 4 jumps to 1dT, 4 jumps to 2 dT, . . . , and 2 jumps to 7 dT. This is a semi-empirical way of correcting for additional biases towards bound molecules.
Max jump (μm)	3	Y	The range of distances to build the histogram of jump lengths. This parameter has to be set so that the tail of the distribution is properly captured. Conversely, a value too high will disturb the fitting, that will be very sensitive to this potentially noisy tail.

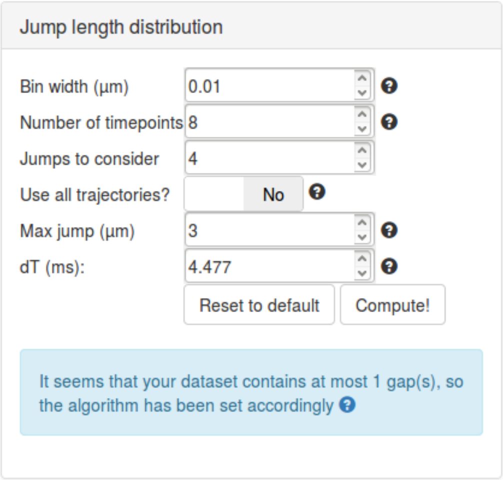

Figure 10: Jump length distribution parameters.

This is the main view of Spot-On, so it is useful to spend a little bit of time to get familiarized with the various options (Figure 11).

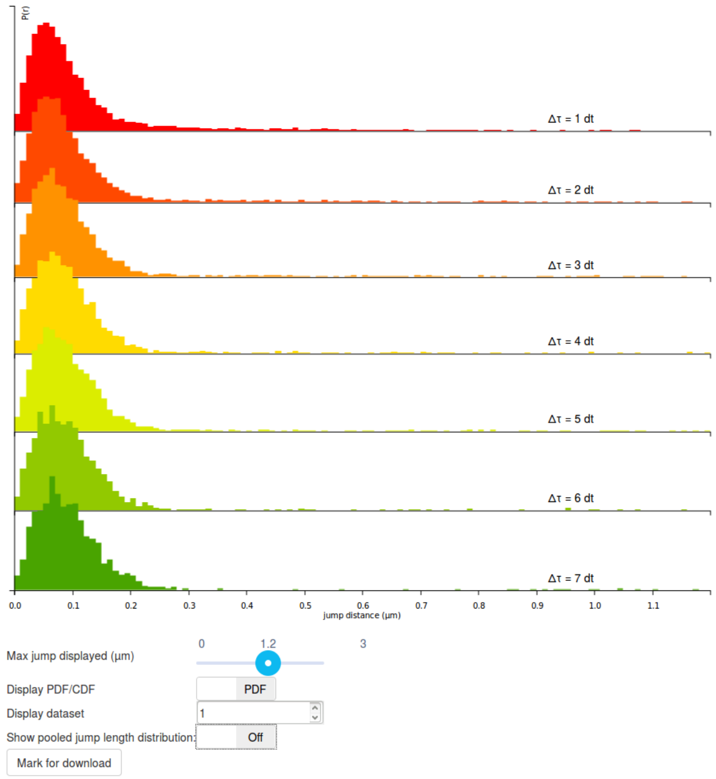

Figure 11: Jump length distribution plot.

2.6.2 Step 5: play with the display options

The graph displayed should be read as follows:

- Each row corresponds to a jump length distribution evaluated at a given Δt. Since short trajectories are more frequent than long trajectories, higher Δt histograms tend to be less populated and appear less smooth (or more "noisy"). The number of rows is determined by the "Number of timepoints" parameter.

- The jump length distribution is computed for values ranging between 0 μm and 3 μm (this corresponds to the "Max Jump" parameter). However, by default, only the first 1.2 μm are initially plotted. To plot the full histogram (or alternatively, to zoom to the origin), the "Max Jump displayed" cursor, located under the plot can be adjusted.

- Then, by default, the jump length distribution is displayed for individual datasets. The displayed dataset is specified in the "Display dataset" box under the plot. It is often useful to take the time to review the jump length distribution of each single acquisition, in order to know which datasets might have to be excluded from further analysis.

- Once individual datasets have been reviewed, it is possible to display the pooled jump length distribution by clicking the "Show pooled jump length distribution" toggle button under the plot. This will compute the distribution for the selected datasets only (in our case, for all the Sox2 datasets). Pooled histograms appear with a hard, black boundary, and the included datasets are displayed under the graph. The updated graph might take a few seconds to render.

The result of all the computations operated by Spot-On are cached. This way, if you enter the same set of parameters as previously, the computation should be almost instantaneous.

2.6.3 Step 6: compare the H2B and Sox2 jump length distributions

Before moving to the fitting, compare the pooled jump length distribution for Sox2 and H2B. To compute the H2B jump length distribution, simply uncheck the Sox2 datasets and select the H2B datasets in the "Dataset selection" area. Then click the *Compute!* button in the "Jump length distribution parameters" box. The two histograms are displayed below. What can you tell from that? Does it match your knowledge of H2B and Sox2?

Answer: When looking at the two histograms side-by-side, the two look very similar at short time scales (up to 200 nm), suggesting that the two proteins show a bound fraction. The dispersion around 70 nm is likely to be characteristic of a combination of localization error (similar at all time scales, from $1\Delta t$ to $7\Delta t$ and of slow diffusion of chromatin (that slowly spreads when looking at higher Δt.

Then, when considering higher distances, the histograms differ significantly, with Sox2 exhibiting a "heavy tail" whereas H2B lacks it. This reflects the fact that H2B is mostly bound whereas Sox2 has a significant freely-diffusing fraction. The modeling approach presented in the next steps

of the tutorial will allow us to better characterize this diffusing state (Figure 12).

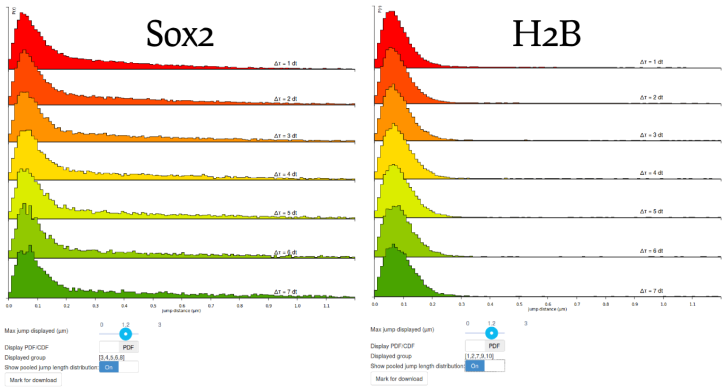

Figure 12: Comparison of the Sox2 and H2B jump length distributions.

2.6.4 Step 7: mark one jump length distribution for download

Before moving to the fitting of the data, let's save this last plot. We will download it later (from the "Download" tab). To do so, click the *Mark for download* button at the bottom of the page. This will prompt a small form where you can enter a name and a description that will be used as a reminder when you download the file. Also, display again the jump length distribution for Sox2 (by selecting the appropriate files and clicking the and *Compute!* button in the "Jump length distribution parameters" box) and save Mark it for download too (Figure 13. We'd get back to these saved analyses later.

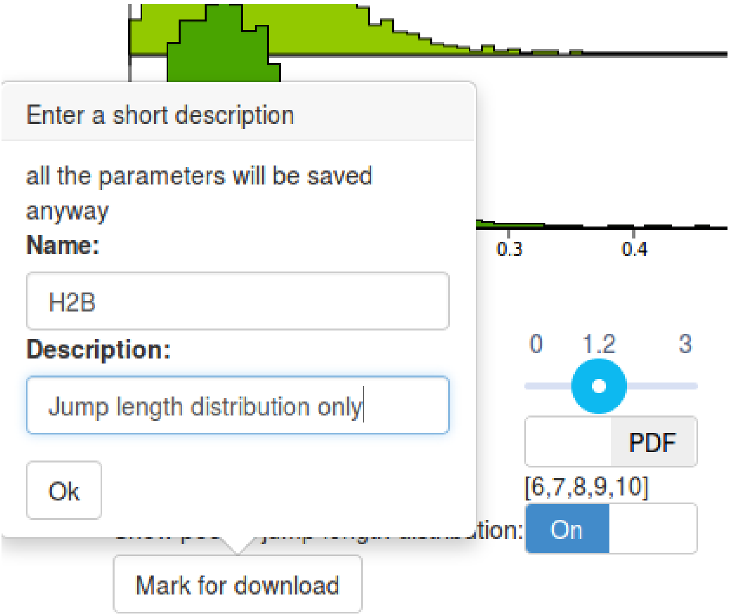

Figure 13: Screenshot of the mark for download box.

2.7 Model fitting

Now that we are familiar with the computation and display of the jump length distribution, let's now move to model fitting!

Spot-On fits the jump length distribution, as defined by the parameters of the "Jump length distribution" box. The fitting parameters are defined in the "Model fitting" box (Table 2.7.1).

2.7.1 Step 8: fit a two-state model to the H2B data

Let's first try to fit a *two-state model*. Click on the picture of the two-state kinetic model (*Bound-Free*, Figure 14). Specific parameter for this model unfold. Let's take a minute to quickly review them (a more detailed description of each parameter is presented in Section Fitting parameters, a short description is shown below).

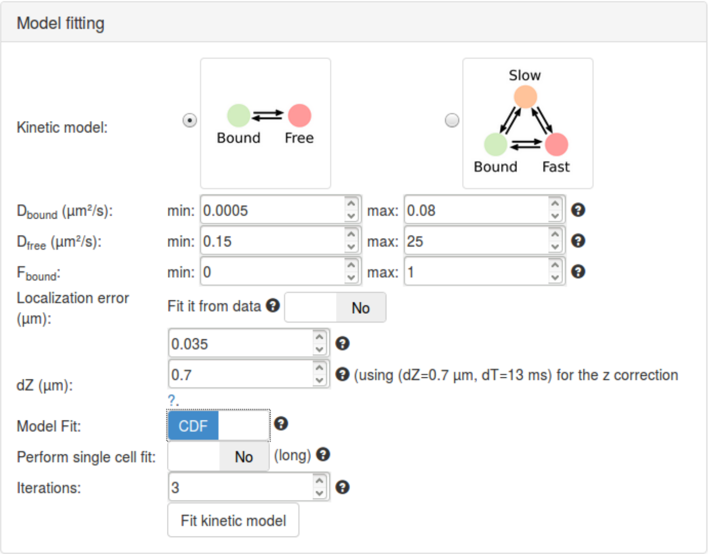

Figure 14: Screenshot of the fitting parameters box.

Having now reviewed the parameters, we can click the *Fit kinetic model* button. A "spinning wheel" will appear next to the button while the fit is being performed and will get displayed when the fit completes.

Parameter	Value	Default?	Description/Comments
Kinetic model	2-state model	N	
Dbound ($\mu m^2/s$)	[0.005, 0.8]	Y	The range of diffusion coefficients for the bound fraction. It is based on a wide plausible range of chromatin diffusion coefficients.
Dfree ($\mu m^2/s$)	[0.15, 25]	Y	The range of diffusion coefficients for the free fraction. These numbers encompass a wide range of free-diffusion coefficients. Note that for diffusion coefficients > 10 $\mu m^2/s$, motion blurring can become a very important issue.
Fbound	[0,1]	Y	The range for the fraction bound.
Localization error (μm)	0.035	Y	See How to measure the localization error? below.
dZ (μm)	0.7	Y	The estimated detection range in z.
Model Fit	CDF	N	Select whether the model will fit the jump length distribution (that is the probability density function, PDF), or the cumulative jump length distribution (CDF)
Perform single cell fit	No	Y	If “Yes”, each individual dataset will be fit. Since our uploaded files are replicates of the same experiment, we want to pool them together.
Iterations	3	Y	The number of times the solver will independently be initialized.

When adjusting the dZ parameter (in the fitting parameters box), you will notice that the mention next to the dZ box changes. The displayed values relate to precomputed coefficients required to perform the correction for particles moving out of focus (see the “Methods” section). These parameters are termed (a, b) and were precomputed over a grid of depths of field (dZ) and exposure times (dT).
However, even though we tried to be as comprehensive as possible in our simulations to derive (a, b), the condition that matches exactly the acquisition settings might be missing. The displayed parameters represent the closest match of the acquisition parameters (dT, dZ) in our simulated database. For most acquisitions setup, the closest precomputed value lies within 0.5 ms and 100 nm of the empirical value (Figure 15).

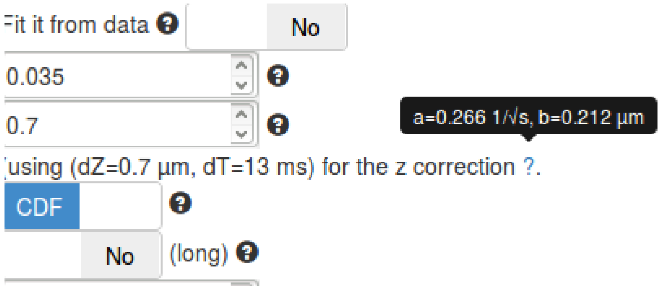

Figure 15: screenshot of the z correction box.

It is important to make sure that the set of displayed parameters is not too far from the real acquisition settings, else, the computed z correction might be biased.

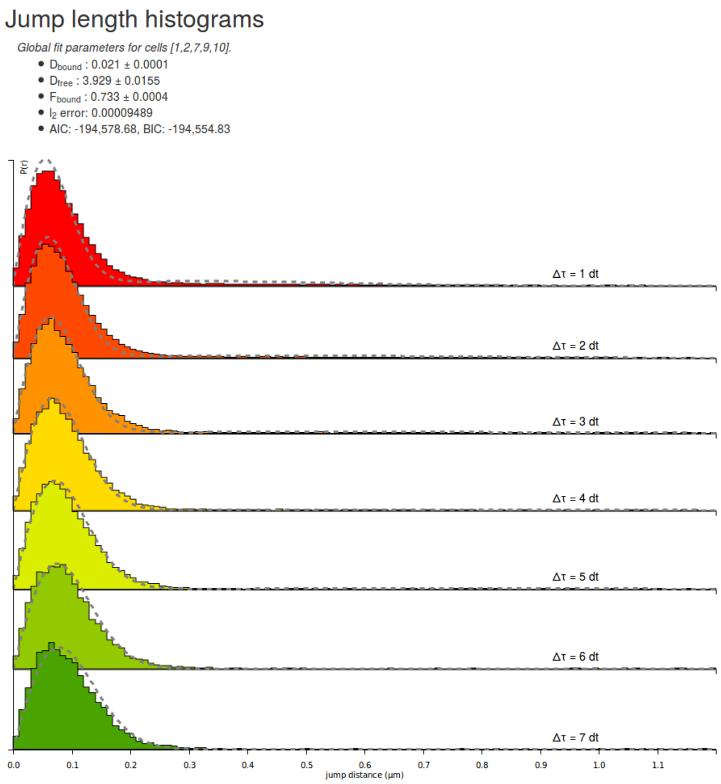

Figure 16: 2-state fitting result of the H2B datasets.

Let's take some time to quickly look at the parameters returned by the fitting routine (Figure 16) for the H2B datasets. Note that due to different initialization values, the returned parameter can differ from execution to execution (Table 2.7.1).

Parameter	Value
Dbound	0.021 $\mu m^2/s$
Dfree	3.929 $\mu m^2/s$
Fbound	0.733
l2 error	0.00009489
AIC	-194578
BIC	-194554

A few comments arise. First, the estimated fraction bound is about 70 %, which is expected from a strongly DNA-associated protein such as H2B.

The associated coefficient with the bound population is close to zero (0.021 μm^2/s) whereas the diffusion coefficient for the free population (3.93 μm^2/s) matches previous knowledge of the dynamics of the protein.

Furthermore, the ℓ_2 error (the mean square error) is $< 10^{-4}$, which can be considered as acceptable (note that this value is not a hard limit and depends on several parameters, including the bin width and the max jump parameters), even though significant misfit appear at low and high Δt: at $1\Delta t$, the fitted distribution is fading faster than the empirical one, whereas at $7\Delta t$, the opposite effect happens. This might be a sign that the protein of interest exhibits anomalous diffusion, or more generally that the model does not fully explain the dynamics of the molecule.

Finally, the AIC (Akaike Information Criterion) and BIC (Bayesian Information Criterion) criteria are provided to allow model comparison. These are two criteria that can be used to compare models and to get hints about which model fits the data best while penalizing for the number of parameters, in order to avoid overfitting.

More specifically, the 3-state model provided by Spot-On has more free parameters than the two-state model (two extra parameters: the "slow" diffusion coefficient and the fraction of the slow-moving fraction). This additional degrees of freedom almost always a better fit than the 2-state model. The AIC and BIC criteria take this difference in the number of parameters and establish a trade-off between the quality of fit (that increases with the number of model parameters) and the number of parameters, in our case penalizing the possible overfitting of the 3-state model.

Although these criteria are useful when comparing the fit of one dataset compared to various models, they cannot be used to assess the quality of fit per se.

2.7.2 Step 9: mark the plot for download

Then, we can save the displayed fit by clicking the *Mark for download* button.

2.7.3 Step 10: fit the Sox2 dataset with a two-state model

We can now proceed similarly to derive the fit for the Sox2 datasets. The resulting fit is shown below, next to a fit using a three-state model. Notice in this plot that significant misfit occurs: at high Δt the model estimates predicts that the bound fraction should have bigger displacements than what

actually is. This characterizes a model mismatch and suggest the use of a three-state model.

2.7.4 Step 11: fit a three-state model

Finally, we can now see how the quality of the fit increases by running the fit again, but with a 3-state model. Select the 3-state model icon (Slow-Bound-Fast) on the "Model fitting" box. New parameters appear, very similarly as with the two-state model. We will leave the parameters to their default values, except for the CDF fit. Then click the *Fit kinetic model* button and wait a until the fitting completes (Figure 17). Observe how the quality of fit evolves and the parameters and estimated fractions between the two-states (Table 2.7.4) and the three-states fit (Table 2.7.4).

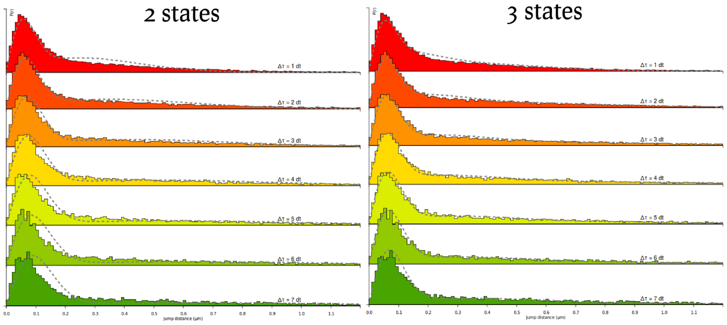

Figure 17: Comparison of 2-state and 3-state fitting for Sox2 datasets.

2-state model

Parameter	Value
Dbound	0.030 $\mu m^2/s$
Dfree	2.410 $\mu m^2/s$
Fbound	0.340
l2 error	0.00039589
AIC	-164571
BIC	-164547

3-state model

Parameter	Value
Dbound	0.012 μm^2/s
Dslow	0.595 μm^2/s
Dfast	4.016 μm^2/s
Fbound	0.256
Fslow	0.258
l2 error	0.00014930
AIC	-185061
BIC	-185021

Based on the information criteria, it is clear that the 3-state model provides a better fit to the data, even when penalizing for the number of parameters.

About model selection. Be careful when interpreting a 3-state model. Indeed, although a two-state model usually appears robust to model mismatch, a 3-state model can fit a wide range of distributions, and the estimated coefficients might be model specific. For instance, the model can invoke a third component to explain what actually is anomalous diffusion.

2.7.5 Step 12: compare the two-state fits of H2B and Sox2 datasets

Let's then fit the H2B data with a two-state model, as described in Step 10 for Sox2 (make sure that you select the right datasets before clicking the Fit button). Once the fit has completed, compare the fitted coefficients between the two proteins (Table 2.7.5 & 2.7.5).

Sox2

Parameter	Value
Dbound	0.030 μm^2/s
Dfree	2.410 μm^2/s
Fbound	0.340

H2B

Parameter	Value
Dbound	0.023 μm^2/s
Dfree	3.84 μm^2/s
Fbound	0.70

Notice that the bound diffusion coefficient are very similar, likely reflecting the diffusion coefficient of DNA/chromatin itself, while the free diffusion coefficients are different, and are likely to reflect different exploration modes of the two proteins. Also notice that the fraction bound are widely different: whereas H2B is mostly bound (70%), Sox2 appears mostly free.

2.8 Download

2.8.1 Step 13: download the marked analyses

Finally move to the "Download" tab, where all the analyses we marked for download are stored. The view should look as in (Figure 18).

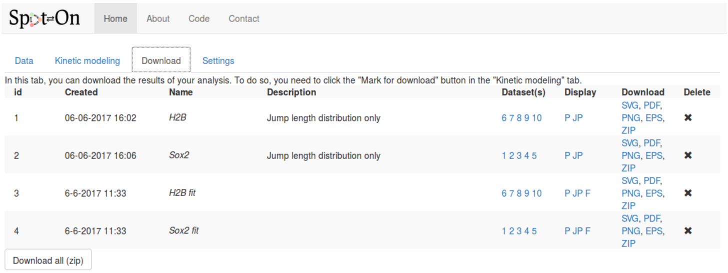

Figure 18: Overview of the download page.

For each analysis marked for download, the following fields are displayed, in addition to the time of the analysis and the name and description we provided in the previous tab (Table 2.8.1).

Column	Description
Name & description	The name & description we provided in the previous tab.
Datasets	The list of datasets included for this plot. Hovering over the numbers displays the full name and description of the dataset.
Display	Descriptor corresponding to the type of plot displayed. Hover over the descriptor to see a short description:<br>P: display of the probability density function,<br>JP: display the pooled jump length distribution,<br>F: pooled fit displayed
Download	Download the corresponding analysis in various formats. The ZIP archive contains all the formats, the raw data, the display parameters and the fitted coefficients (if any).
Delete	To delete this analysis.

3 Online sections

This document only contains an abbreviated version of the documentation for Spot-On. The complete manual for the latest version is available online at:

`https://spoton.berkeley.edu/SPTGUI/docs/latest`

In addition to this tutorial, the following sections are available:

- Software reference
  - Input formats
  - Descriptors of imported datasets
  - Dataset statistics
  - Jump length distribution computation parameters
  - Fitting parameters
  - Display parameters
- How to acquire a "good" dataset?
  - Detection – minimizing "motion-blurring"
  - Tracking – minimizing tracking errors
  - Which datasets are appropriate for Spot-On?
- Methods
  - Outline of the method
  - Derivation of the two states kinetic model
  - Generalization to a 3-state model
  - Assumptions of the approach
  - Numerical implementation
  - References
- Code
- Datasets

  - Construction of the cell lines
  - Data acquisition
  - ExpA
  - ExpB
  - Data availability

- Frequently asked questions
  - What is Spot-On?
  - What is not Spot-On?
  - What tracking software to use?
  - What types of input does Spot-On accept?
  - My input format does not seem to be supported, what can I do?
  - What limits the length of trajectories?
  - Are you just fitting a two-exponential model?
  - How to measure the localization error?
  - How to measure the axial detection range?
  - How fast is Spot-On?
  - I'm afraid of uploading my dataset to your server. Is there an offline version?
  - Is there a command-line version?
  - What technology is used by Spot-On?
  - Is there a Matlab® version?
  - What license uses Spot-On?
  - I have a question
  - How do you handle privacy?
  - How to cite Spot-On?
  - How to contact you?
  - I found a bug, how can I report it?

References

[Grimm et al., 2015] Grimm, J. B., English, B. P., Chen, J., Slaughter, J. P., Zhang, Z., Revyakin, A., Patel, R., Macklin, J. J., Normanno, D., Singer, R. H., Lionnet, T., and Lavis, L. D. (2015). A general method to improve fluorophores for live-cell and single-molecule microscopy. *Nature Methods*, 12(3):244–250. 00066.

[Mazza et al., 2012] Mazza, D., Abernathy, A., Golob, N., Morisaki, T., and McNally, J. G. (2012). A benchmark for chromatin binding measurements in live cells. *Nucleic Acids Research*, 40(15):e119–e119.

[Shinkai et al., 2016] Shinkai, S., Nozaki, T., Maeshima, K., and Togashi, Y. (2016). Dynamic nucleosome movement provides structural information of topological chromatin domains in living human cells. *PLOS Computational Biology*, 12(10):e1005136.
