# Supplementary material for: Robust model-based analysis of single-particle tracking experiments with Spot-On
Source: eLife. 2018 Jan 4;7:e33125. doi: 10.7554/eLife.33125 (PMC5809147; doi:10.7554/eLife.33125)
Supplement: Transparent reporting form [file elife-33125-transrepform.docx]

***eLife’s* transparent reporting form**

We encourage authors to provide detailed information *within their submission* to facilitate the interpretation and replication of experiments. Authors can upload supporting documentation to indicate the use of appropriate reporting guidelines for health-related research (see [EQUATOR Network](http://www.equator-network.org/%20)), life science research (see the [BioSharing Information Resource](https://biosharing.org/)), or the [ARRIVE guidelines](http://www.plosbiology.org/article/info:doi/10.1371/journal.pbio.1000412) for reporting work involving animal research. Where applicable, authors should refer to any relevant reporting standards documents in this form.

If you have any questions, please consult our Journal Policies and/or contact us: [editorial@elifesciences.org](mailto:editorial@elifesciences.org).

**Sample-size estimation**

- You should state whether an appropriate sample size was computed when the study was being designed
- You should state the statistical method of sample size computation and any required assumptions
- If no explicit power analysis was used, you should describe how you decided what sample (replicate) size (number) to use

Please outline where this information can be found within the submission (e.g., sections or figure legends), or explain why this information doesn’t apply to your submission:

The nature of errorbars (e.g. standard deviation vs. standard error) is listed in the figure legend for the relevant Figure. Explanation of replicates and the number of cells is listed in the figure legend as well as Materials and Methods. Finally, all the raw data supporting this paper is freely available at Zenodo (<https://zenodo.org/record/834781>) and each experiment and replicate is clearly described there as well. We did not use a statistical method to determine sample size, but rather made sure that more than enough cells were present for each replicate to achieve highly consistent results between replicates.

**Replicates**

- You should report how often each experiment was performed
- You should include a definition of biological versus technical replication
- The data obtained should be provided and sufficient information should be provided to indicate the number of independent biological and/or technical replicates
- If you encountered any outliers, you should describe how these were handled
- Criteria for exclusion/inclusion of data should be clearly stated
- High-throughput sequence data should be uploaded before submission, with a private link for reviewers provided (these are available from both GEO and ArrayExpress)

Please outline where this information can be found within the submission (e.g., sections or figure legends), or explain why this information doesn’t apply to your submission:

For experiments in Figure 4, replicate refers to experiments performed on different days using the same cell line. This is also explaining in the Figure Legend and Materials and Methods section. Finally, all the raw data supporting this paper is freely available at Zenodo (<https://zenodo.org/record/834781>) and each experiment and replicate is clearly described there as well.

**Statistical reporting**

- Statistical analysis methods should be described and justified
- Raw data should be presented in figures whenever informative to do so (typically when N per group is less than 10)
- For each experiment, you should identify the statistical tests used, exact values of N, definitions of center, methods of multiple test correction, and dispersion and precision measures (e.g., mean, median, SD, SEM, confidence intervals; and, for the major substantive results, a measure of effect size (e.g., Pearson's r, Cohen's d)
- Report exact p-values wherever possible alongside the summary statistics and 95% confidence intervals. These should be reported for all key questions and not only when the p-value is less than 0.05.

Please outline where this information can be found within the submission (e.g., sections or figure legends), or explain why this information doesn’t apply to your submission:

We did not calculate any p-values and did not perform any statistical tests. Information about sample sizes is available together with the raw trajectory data at Zenodo (<https://zenodo.org/record/834781>)

(For large datasets, or papers with a very large number of statistical tests, you may upload a single table file with tests, Ns, etc., with reference to sections in the manuscript.)

**Group allocation**

- Indicate how samples were allocated into experimental groups (in the case of clinical studies, please specify allocation to treatment method); if randomization was used, please also state if restricted randomization was applied
- Indicate if masking was used during group allocation, data collection and/or data analysis

Please outline where this information can be found within the submission (e.g., sections or figure legends), or explain why this information doesn’t apply to your submission:

There was no group allocation in this study.

**Additional data files (“source data”)**

- We encourage you to upload relevant additional data files, such as numerical data that are represented as a graph in a figure, or as a summary table
- Where provided, these should be in the most useful format, and they can be uploaded as “Source data” files linked to a main figure or table
- Include model definition files including the full list of parameters used
- Include code used for data analysis (e.g., R, MatLab)
- Avoid stating that data files are “available upon request”

Please indicate the figures or tables for which source data files have been provided:

All raw data and all code is fully open-source and freely available. The experimental data is available at: <https://zenodo.org/record/834781>; The simulations are available in Matlab format at: <https://zenodo.org/record/835541>; The simulations are available in CSV format at: <https://zenodo.org/record/834787>; And supplementary software used for MSD_i_ and vbSPT analysis as well as for generating the simulated data at: <https://zenodo.org/record/835171>

The web-interface can be found at: [https://SpotOn.berkeley.edu](http://spoton.berkeley.edu/). All raw code is available at GitLab: <https://gitlab.com/tjian-darzacq-lab>. The web-interface code can be found at <https://gitlab.com/tjian-darzacq-lab/Spot-On>; the Matlab command-line version of Spot-On can be found at: <https://gitlab.com/tjian-darzacq-lab/spot-on-matlab>; the Python command-line version of Spot-On can be found at <https://gitlab.com/tjian-darzacq-lab/Spot-On-cli>; finally, the SPT simulation code (simSPT) can be found at: <https://gitlab.com/tjian-darzacq-lab/simSPT>.

Finally, we note that the raw data is available as SPT trajectories – the underlying raw movies (nd2 format) represent ~2 TB of data and are available upon request.
